# Functional differences in echolocation call design in an adaptive radiation of bats

**DOI:** 10.1002/ece3.8296

**Published:** 2021-11-02

**Authors:** Leith B. Leiser‐Miller, Sharlene E. Santana

**Affiliations:** ^1^ Department of Biology University of Washington Seattle Washington USA; ^2^ Burke Museum of Natural History and Culture University of Washington Seattle Washington USA

**Keywords:** acoustics, Chiroptera, diet, neotropics, Phyllostomidae, sensory ecology

## Abstract

All organisms have specialized systems to sense their environment. Most bat species use echolocation for navigation and foraging, but which and how ecological factors shaped echolocation call diversity remains unclear for the most diverse clades, including the adaptive radiation of neotropical leaf‐nosed bats (Phyllostomidae). This is because phyllostomids emit low‐intensity echolocation calls and many inhabit dense forests, leading to low representation in acoustic surveys. We present a field‐collected, echolocation call dataset spanning 35 species and all phyllostomid dietary guilds. We analyze these data under a phylogenetic framework to test the hypothesis that echolocation call design and parameters are specialized for the acoustic demands of different diets, and investigate the contributions of phylogeny and body size to echolocation call diversity. We further link call parameters to dietary ecology by contrasting minimum detectable prey size estimates (MDPSE) across species. We find phylogeny and body size explain a substantial proportion of echolocation call parameter diversity, but most species can be correctly assigned to taxonomic (61%) or functional (77%) dietary guilds based on call parameters. This suggests a degree of acoustic ecological specialization, albeit with interspecific similarities in call structure. Theoretical MDPSE are greatest for omnivores and smallest for insectivores. Omnivores significantly differ from other dietary guilds in MDPSE when phylogeny is not considered, but there are no differences among taxonomic dietary guilds within a phylogenetic context. Similarly, predators of non‐mobile/non‐evasive prey and predators of mobile/evasive prey differ in estimated MDPSE when phylogeny is not considered. Phyllostomid echolocation call structure may be primarily specialized for overcoming acoustic challenges of foraging in dense habitats, and then secondarily specialized for the detection of food items according to functional dietary guilds. Our results give insight into the possible ecological mechanisms shaping the diversity of sensory systems, and their reciprocal influence on resource use.

## Graphical abstract

Most bat species use echolocation for navigation and foraging, but which and how ecological factors shaped echolocation call diversity remains unclear for the most diverse clades, including the adaptive radiation of neotropical leaf‐nosed bats (Phyllostomidae). We report that phyllostomid echolocation call structure may be primarily specialized for overcoming acoustic challenges of foraging in dense habitats, and then secondarily specialized for the detection of food items according to functional dietary guilds.

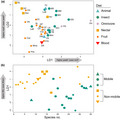

## 1 INTRODUCTION

For many animals, sound perception is vital for conducting ecological tasks, and bats are exceptional in their sophisticated use of echolocation for spatial orientation, navigation, communication, and foraging (Geipel et al., 2013; Jones & Siemers, 2011; Jung et al., 2014; Schnitzler et al., 2003; Siemers & Schnitzler, 2004). As diverse as the functions of echolocation are the factors that have been associated with variation in echolocation call structure, including phylogeny, sociality, diet, and habitat (Jones & Siemers, 2011; Puechmaille et al., 2014; Russ et al., 2005; Schuchmann et al., 2012; Voigt‐Heucke et al., 2010; Wilkinson & Wenrick Boughman, 1998). Among these factors, foraging ecology (e.g., foraging habitat and diet) is a strong predictor of call structure in bats (Jones, 1999). However, assessments of call structure differences across guilds are usually based on broadly defined foraging categories (e.g., aerial‐hawking vs. gleaning bats; Jones, 1999) comparing ecologically distinct families. Furthermore, the call characteristics that are typically compared, such as the distinction between constant frequency (CF) and frequency‐modulated (FM) calls, represent coarse assessments of echolocation calls. Less is known about call structure differences at finer resolution within families of bats, particularly those that are trophically diverse and/or have calls that are difficult to record (e.g., “whispering” bats, high‐flying bats).

Phyllostomidae (Neotropical leaf‐nosed bats) are an adaptive radiation of over 200 species (Dumont et al., 2012; Rossoni et al., 2017). Phyllostomids exhibit the greatest dietary diversity of any bat family, including insectivory, sanguinivory, animalivory, nectarivory, omnivory, and frugivory (Dumont et al., 2012; Rex et al., 2010). Previous studies have implicated craniodental morphology, biting behavior, and performance traits (e.g., foreshortened rostrum, unilateral molar bites, high bite force; Dumont et al., 2012; Santana & Dumont, 2009; Santana et al., 2010) as adaptations to novel prey in phyllostomids. However, dietary ecology has not only shaped traits for prey processing; phyllostomids with different diets also exhibit sensory biases; and these appear to have played an important role in the dietary adaptive radiation of these bats (Gonzalez‐Terrazas, Martel, et al., 2016; Hall et al., 2021; Jones et al., 2013; Kalko & Condon, 1998; Kürten & Schmidt, 1982; Müller et al., 2009; Safi & Siemers, 2010; Thies et al., 1998). Still, less is understood about whether and how the phyllostomid echolocation system (e.g., call parameters, behavior, morphology of sensory structures) evolved in tandem with their dietary radiation.

Broadly, phyllostomids are narrow‐space foragers that primarily feed in the forest understory or canopy (Wilson & Reeder, 2005); thus, their main echolocation task is short‐range object detection in highly cluttered acoustic spaces (e.g., overcoming acoustic masking echoes from foliage and other obstacles, Schnitzler & Kalko, 2001). Traditionally, phyllostomids have been considered “whispering” bats because they typically emit highly directional calls at lower intensities than species in other bat families (Griffin, 1958), although research has shown that some species may be capable of calling at higher intensities (Brinkløv et al., 2009). Phyllostomids are underrepresented in comparative acoustic studies because of limitations associated with recording low‐intensity, high‐frequency calls in the hot, humid, and densely forested environments most species inhabit (Griffin, 1971). While previous studies have been largely qualitative and deemed phyllostomid call structure as relatively uniform across species, there is also evidence that their calls might be more diverse than previously thought (Gessinger et al., 2019; Kalko, 2004; Yoh et al., 2020). Therefore, quantitative analyses of larger datasets collected in a systematic fashion have the potential to reveal that phyllostomid calls are associated with their dietary specializations. In fact, some phyllostomids seem to deviate from allometric call parameter patterns exhibited by other animals (e.g., bats; Hipposideridae, Rhinolophidae, Emballonuridae, Vespertilionidae, and Molossidae; Jones, 1999; frogs, Ryan, 1985; birds, Martin et al., 2011; Ryan & Brenowitz, 1985), suggesting that phylogeny and/or dietary ecology may contribute to echolocation call diversity in these bats (Jacobs et al., 2007).

Echolocation call parameters have specific functions in shaping the acoustic field of view. Frequency is particularly important for encoding audible echo reflection (Møhl, 1988; Pye, 1993), range accuracy (Stamper et al., 2009), and detecting targets against forest clutter (Bates et al., 2011). For the detection of a specific object, such as a prey item, acoustic theory predicts that spheres reflect weak echoes if their circumference is smaller than the wavelength of the impinging sound (Pye, 1993). Ensonification experiments further suggest that small insects may reflect sound in a similar way to spheres, and therefore, bats must use high frequencies (short wavelengths) to obtain an audible echo from small insects (Møhl, 1988; Safi & Siemers, 2010). Previous work has further demonstrated emitted call frequency is related to prey size in some vespertilionid bat species, supporting the hypothesis that call frequency and prey size can be functionally linked (Thomas et al., 2004). To date, it is unknown if this basic relationship exists in phyllostomids bats.

Here, we report a dataset spanning 21 genera, 35 species, and all dietary guilds of phyllostomid bats. We use these data to quantify the structure of phyllostomid echolocation calls (both time‐ and frequency‐linked parameters) and conduct phylogenetic analyses to test the hypothesis that the design and parameters of phyllostomid echolocation calls are specialized to the acoustic demands imposed by different diets. We also explore if body size and phylogeny underlie diversity in call structure across species, and further link call parameters and dietary ecology by calculating and comparing estimates of minimum detectable prey sizes across species. Given patterns reported for other families of bats (Jones, 1999), we predict that call parameters (see Table 1 for definitions) will not scale with body size in phyllostomids. We also predict species within the same dietary guild will have similar call parameters, independent of phylogenetic relatedness (see Table 2 for specific predictions), and dietary guilds will differ in their estimated minimum detectable prey size. Specifically, insectivores will have the smallest detectable prey size (i.e., due to highest call frequency and shortest wavelength), and omnivores will have the largest detectable prey size (i.e., due to lowest call frequency and longest wavelength) because these bats forage for larger prey (e.g., vertebrates, large fruit; Kalko & Condon, 1998) and use other senses besides echolocation for prey detection.

**TABLE 1:** Definition and functional significance of call parameters. Within each call parameter group (Par. groups) are the specific call parameters (Call specific) measured in this study, along with their function, predictor traits, and associated citations for functions and predictors

Par. groups	Call specific	Function	Predictor traits	Citation
Harmonics		Distinguish clutter echoes from target echoes	Unknown	Simmons et al. (1975), Bates et al. (2011)
Frequency		Influences acoustic field of view (i.e., sonar beam width), influences resolution of acoustic image		Neuweiler (2000), Bates et al. (2011), Fenton et al. (2016)
	Minimum frequency	Low values: increase range detection, increase beam width, decrease resolution	Unknown	Neuweiler (2000), Bates et al. (2011), Fenton et al. (2016)
	Maximum frequency	High values: decrease range detection, decrease beam width, increase resolution, and target discrimination	Unknown	Neuweiler (2000), Bates et al. (2011), Fenton et al. (2016)
	Peak frequency	Reflects the highest energy, most critical for determining field of view	Body size	Jones (1999), Bates et al. (2011)
Bandwidth		High values: better temporal resolution and accuracy in range detection	Unknown	Simmons et al. (1975), Denzinger and Schnitzler (2013), Fenton et al. (2016)
	Narrow frequency band	High values: encode information about small frequency changes produced by fluttering insects Low values: better for detecting larger objects at longer distances	Foraging habitat	Fenton et al. (2016)
	Broad frequency band	High values: reduce masking effects for foraging within clutter (particularly on insects), improves lower resolution limit	Foraging habitat, diet	Siemers and Schnitzler (2004), Boonman and Ostwald (2007), Denzinger and Schnitzler (2013)
Duration		Low values: optimize the resolution of target distance and range accuracy, increases the signal overlap‐free window zone (i.e., no echo interference) High values: decrease the acoustic overlap‐free window, making is difficult to distinguish outgoing from incoming call information and clutter echoes from target echoes	Foraging habitat	Simmons et al. (1975), Denzinger and Schnitzler (2013), Fenton et al. (2016)

**TABLE 2:** Hypotheses and predictions for specific call parameters (Call specific) within each call parameter group (Par. groups), as well as predicted dietary guild to exhibit each prediction

Par. groups	Call specific	Prediction	Dietary guild (taxonomic/functional)
Harmonics		Species that forage on cryptic prey in dense clutter will have a higher number of harmonics	Insectivore/mobile‐evasive
Frequency	Minimum frequency	Species that forage over long distances and on larger prey will have lower values of minimum frequency	Sanguinivore, omnivore/non‐mobile‐non‐evasive
	Maximum frequency	Species that forage on highly cryptic prey such as insects on leaves or vertebrates will have higher values of maximum frequency	Insectivore, animalivore/mobile‐evasive
	Peak frequency	Similar within and different between dietary guilds, as each guild experiences a different set of foraging challenges	–
Bandwidth	Narrow frequency band	Species that forage over longer distances and on larger prey items, or prey items separated from leaves (i.e., fruits and flowers), will exhibit lower values of bandwidth	Omnivore, sanguinivore/non‐mobile‐non‐evasive
	Broad frequency band	Species that forage for prey in dense clutter and where prey is hidden in clutter will exhibit higher values of bandwidth	Insectivore/non‐mobile‐non‐evasive
Duration		Species that heavily rely on echolocation to detect and locate prey on leaf background will have shorter duration of calls	Insectivore/mobile‐evasive

## 2 MATERIALS AND METHODS

### 2.1 Acoustics

We used mist nets to collect free‐ranging bats at Palo Verde National Park, Guanacaste, Costa Rica, and La Selva Biological Station, Sarapiquí, Costa Rica from 2015 to 2018, through the months of January–March and July–December. We recorded release calls from 153 individuals spanning 21 genera and 35 species (Table S1) using an Avisoft UltraSoundGate 116H recording interphase with an Avisoft‐Bioacoustics CM16/CMPA externally polarized condenser microphone, at 375 kHz sampling rate and 16‐bit recording. While these settings resemble those used by previous studies and should be adequate to resolve the call parameters of most phyllostomids in our sample, we acknowledge that they may result in underestimation of frequencies for species with broadband calls that start above 140 kHz (e.g., *Glossophaga soricina*, *Micronycteris microtis*; Geipel et al., 2013; Knörnschild et al., 2010; Simon et al., 2014). To record calls, we held each bat in hand, placed a microphone approximately 15 cm from its face, and then released the bat away from environmental clutter while recording the calls emitted as it flew away. We measured call parameters for 2–12 individuals per species except for six species that were rare or difficult to capture at our study localities, for which we only recorded one individual per species (Table S1). All collecting and handling procedures were approved by the University of Washington's Institutional Animal Care and Use Committee (protocol# 4307‐01).

**FIGURE 1:**
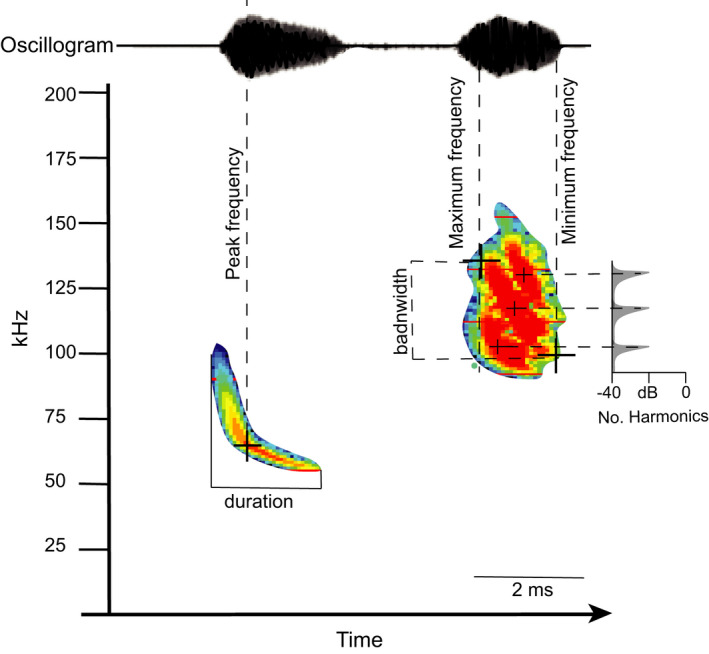
Schematic of spectrogram of *Hylonycteris underwoodi* (left) and *Platyrrhinus helleri* (right) illustrating measurement points of echolocation call parameters used in the analyses. Oscillogram (top) represents amplitude of calls. Duration of the call is calculated as the length of the call at an amplitude above −20 dB relative to the maximum amplitude of the call, maximum frequency is taken at maximum amplitude at the start of the call, minimum frequency is taken at maximum amplitude at the end of the call, peak frequency is the maximum frequency over the entire call, bandwidth is calculated as the difference between the maximum and minimum frequency over the entire call, and number (No.) of harmonics is taken as the number of peaks with amplitude greater than −20 dB relative to the maximum amplitude of the individual spectrum

We analyzed release calls using Avisoft SASLabPro v. 5.2.12 (Avisoft Bioacoustics, Berlin, Germany). To optimize both frequency and temporal resolution, we set the frequency resolution parameters for the spectrogram at a fast Fourier transform (FFT; Brigham, 1988) length of 256, 100% frame size, with a flattop window. We also set the temporal resolution for the spectrogram with a window overlap of 93.75%. We then set the automatic measurements algorithm to take measurements of call duration, peak frequency, maximum frequency, minimum frequency, bandwidth, and number of harmonics at appropriate locations for each call within each file (Figure 1). We manually inspected each call classified by the automatic measurements algorithm to ensure accuracy in element detection. If ultrasonic background noise above −20 dB was influencing measurements, we manually erased this noise from the spectrogram and recalculated measurements. To further reduce the influence of high‐intensity, low‐frequency sounds generated by background noise, we filtered all call files with a high‐pass band filter set at 20 kHz, except for the calls of *Phyllostomus hastatus*. This species had calls with a lower minimum frequency than other phyllostomids, so we set a high‐pass band filter at 10 kHz. To determine valid calls in a recorded file, we set element separation at a hold time of 2 ms (i.e., within the range of call duration for phyllostomids, Brigham et al., 2002; Jennings et al., 2004; Kalko & Condon, 1998; Thies et al., 1998; Weinbeer & Kalko, 2007), with the exception of *Centurio senex*, for which we set a hold time of 10 ms because of the extended duration of this species’ call.

We averaged call sequences per individual (a minimum of 5 calls per file) and calculated means and standard deviations of each measured parameter. We also report the range of each call parameter in the form of the maximum and minimum value recorded (Table S1). To estimate the theoretical, minimum prey size detectable given an emitted frequency, we used the equation *λ* = *v*/*f* (Yang, 2010), where *λ* is the wavelength, v is the wave velocity (speed of sound in air), and *f* is the frequency. For speed of sound, we used 347 m/s for 25°C and 68% humidity, which reflects average annual environmental conditions of the sites where we recorded calls (sourced from worldweatheronline.com). While numerous other factors such as source level of the call, distance to the prey item, and auditory sensitivity ultimately determine a species’ actual detectable prey size, we hope these theoretical estimates can serve to inform predictions that could be tested in future ecological and experimental studies.

### 2.2 Statistical analyses

First, we tested each call parameter (duration, maximum frequency, minimum frequency, peak frequency, bandwidth, and harmonics) for phylogenetic signal by calculating Pagel's lambda (Freckleton et al., 2004; Pagel, 1992) using a pruned version of the Rojas et al. (2016) phylogeny and the function phylosig from the package “phytools” version 0.6‐60 (Revell, 2012) in RStudio version 1.1.463.

To test if call parameters are predicted by body size, we ran phylogenetic generalized least squares (PGLS) regressions using the function pgls in “phytools” and a pruned version of the Rojas et al. (2016) phyllostomid phylogeny, with individual call parameters as a response variable and forearm length (a standard measure of body size in bats, sourced from the literature; Timm & LaVal, 1998) as a predictor variable. To test if specific call parameters are associated with dietary guild, we grouped species into one of six taxonomic dietary categories: animalivores, insectivores, nectarivores, frugivores, omnivores, and sanguinivores (Giannini & Kalko, 2004). Since these may not reflect the acoustic challenges associated with detecting different food/prey items, and accumulating research suggests that some phyllostomid species may not be restricted to these specific dietary guilds (Clare et al., 2014; Rex et al., 2010), we also tested for differences in call structure between two functional dietary guilds: predators of non‐mobile, non‐evasive prey (nectarivores, frugivores, and sanguinivores), and predators of mobile, evasive prey (omnivores, insectivores, and animalivores). To identify call parameters that differentiate echolocation calls between dietary guilds, we conducted a linear discriminant analysis (LDA) using the function lda from the R package “MASS” version 7.3‐49 (Ripley et al., 2013). We then used the model derived from the LDA to test if echolocation call traits are predictors of a species’ dietary guild.

Finally, to test if differences in call parameters are linked to dietary guilds under an evolutionary context, we used the function sim.char from the R package “geiger” version 2.0.6.1 (Harmon et al., 2019) to simulate the evolution of call parameters on the phylogeny for each level of dietary assignment (i.e., taxonomic categories and functional guilds). Then, to test for differences in minimum detectable prey size estimates among taxonomical and functional dietary guilds, we performed phylogenetic ANOVAs using the function aov.phyl from the R package “geiger” version 2.0.6.1 (Harmon et al., 2019).

## 3 RESULTS

### 3.1 Phylogenetic signal and scaling of call parameters

**FIGURE 2:**
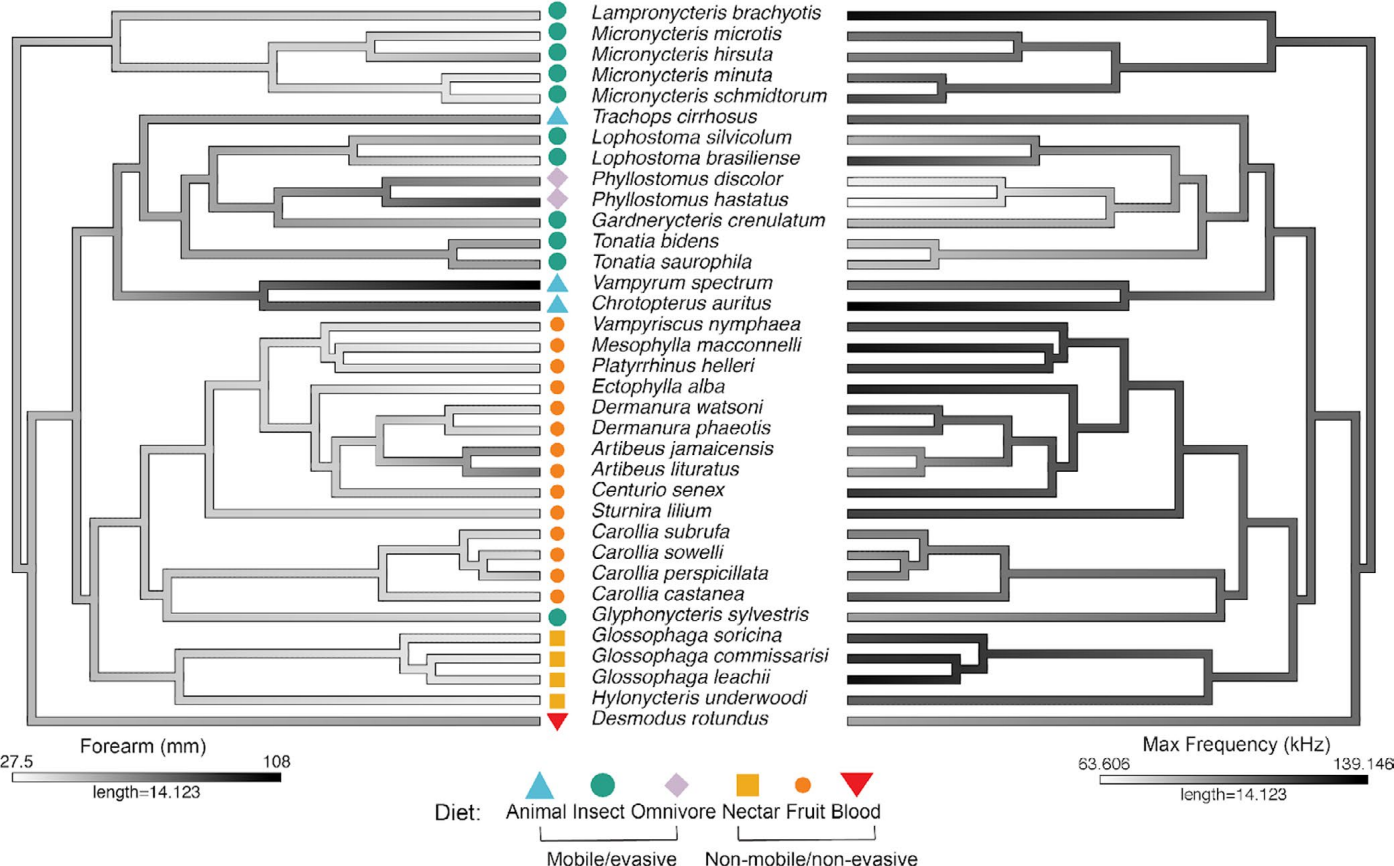
Forearm length (left) and maximum frequency (right) mapped on the phylogeny of the phyllostomid species included in this study. Ancestral character states were estimated using the contmap function in phytools (Revell, 2012) on the Rojas et al. (2016) phylogeny. Taxonomic and functional dietary guilds used in analyses are denoted with symbols

We found that duration (*λ* = 0.74), maximum frequency (*λ* = 1), minimum frequency (*λ* = 0.92), and peak frequency (*λ* = 1) all exhibit a relatively high phylogenetic signal. That is, more closely related species share more similarity in these call parameters (but note they also have similar diet and foraging habitats; Figure 2). Conversely, bandwidth (*λ* = 0.54) and number of harmonics (*λ* = 7.35e−05) exhibit low to negligible phylogenetic signal. We found a significant, negative relationship between forearm length (FL) and maximum echolocation call frequency (PGLS: *b* = −0.4766, *R* ^2^ = 0.2, *p* = .007; Figure 2 and Figure S1), but this body size metric was not a significant predictor of call duration, minimum frequency, peak frequency, number of harmonics, or sweep rate (all *p* > .05).

### 3.2 Discrimination of call structure among dietary guilds

**FIGURE 3:**
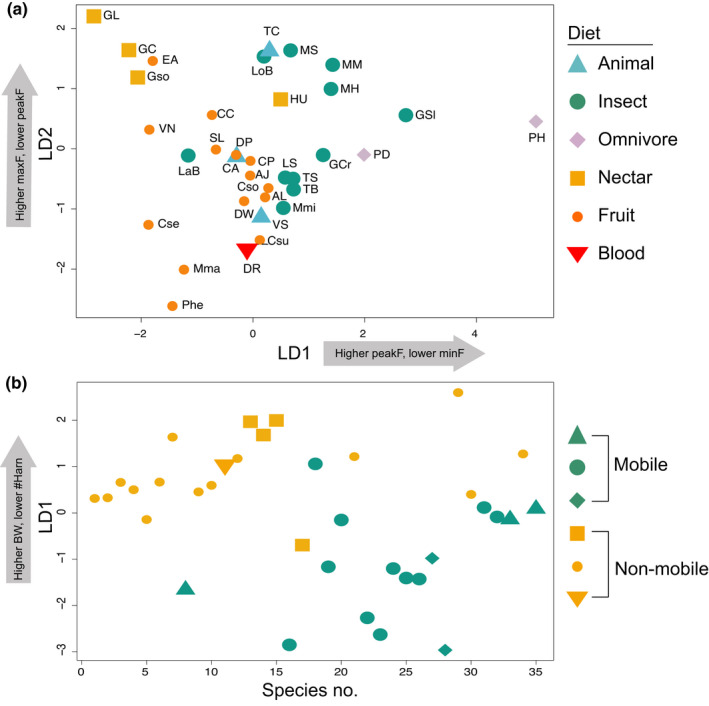
Linear discriminant analysis (LDA) of (a) taxonomically defined dietary guilds and (b) functionally defined dietary guilds. PeakF: peak frequency, minF: minimum frequency, maxF: maximum frequency, BW: bandwidth, #Harm: number of harmonics. Species codes are listed as AJ‐*Artibeus jamaicensis*; AL, *Artibeus lituratus*; CC, *Carollia castanea*; CP, *Carollia perspicillata*; Cso, *Carollia sowelli*; Csu, *Carollia subrufa*; Cse, *Centurio senex*; CA, *Chrotopterus auritus*; DP, *Dermanura phaeotis*; DW, *Dermanura watsonii*; DR, *Desmodus rotundus*; EA, *Ectophylla alba*; GCr, *Gardnerycteris crenulatum*; GC, *Glossophaga commissarisi*; GL, *Glossophaga longirostirs*; Gso, *Glossophaga soricina*; GSl, *Glyphonycteris sylvestris*; HU, *Hylonycteris underwoodi*; LaB, *Lampronycteris brachyotis*; LoB, *Lophostoma brasiliense*; LS, *Lophostoma silvicolum*; Mma, *Mesophylla macconnelli*; MH, *Micronycteris hirsuta*; MM, *Micronycteris microtis*; Mmi, *Micronycteris minuta*; MS, *Micronycteris schmidtorum*; PD, *Phyllostomus discolor*; PH, *Phyllostomus hastatus*; Phe, *Platyrrhinus helleri*; SL, *Sturnira lilium*; TB, *Tonatia bidens*; TS, *Tonatia saurophila*; TC, *Trachops cirrhosus*; VN, *Vampyriscus nymphaea*; VS, *Vampyrum spectrum*

A discriminant analysis for taxonomically defined guilds indicated that the first discriminant axis (LD1, Figure 3a) is characterized by a strong positive loading of peak frequency and a strong negative loading of minimum frequency. This axis primarily separates frugivores and most nectarivores (e.g., *Glossophaga*; −LD1) from most insectivores and omnivores (+LD1). Animalivores and sanguinivores fall between these groups along LD1. Maximum call frequency has a strong positive loading on the second discriminant axis (LD2), while peak frequency has a strong negative loading. There is considerable overlap among guilds along LD2. Each taxonomically defined dietary category includes some species that are outliers with respect to their dietary guild along both LD1 and LD2; specifically, *Lampronycteris brachyotis*, an insectivore, shares more similarities with frugivores; *Hylonycteris underwoodi*, a nectarivore, shares more similarities with insectivores; *Phyllostomus hastatus*, an omnivore, is distinct from all species in call design; and the animalivores *Chrotopterus auritus*, *Vamyprum spectrum*, and *Trachops cirrhosus* are starkly different from each other and more similar to either insectivores or frugivores. Insectivores, except for *L*. *brachyotis*, occupy two different areas of acoustic space, which is primarily driven by differences in peak and minimum frequency (Figure 3a). LDA predictions assigned 61.7% of species to the correct taxonomically defined dietary category (*p* = .01).

When functionally defined guilds are considered, bandwidth has a strong positive loading on the first discriminant axis (+LD1) and a strong negative relationship with number of harmonics (−LD1, Figure 3b). This axis largely separates species feeding on non‐mobile/non‐evasive prey (+LD1) from species feeding on mobile/evasive prey (−LD1). LDA predictions correctly assigned 77.1% of species to the correct functionally defined dietary guild (*p* = .02).

### 3.3 Minimum detectable prey size

**FIGURE 4:**
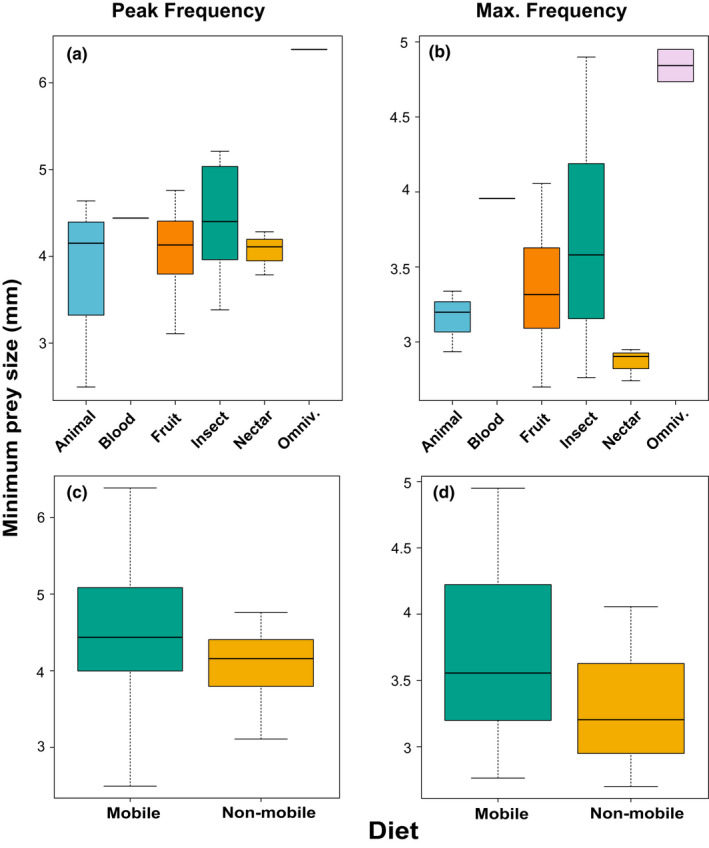
Minimum detectable prey size (mm) across taxonomically defined dietary guilds (top) and functionally defined dietary guilds (bottom), calculated from measured peak frequency (a, c) and maximum frequency (b, d) of echolocation calls

We estimated the minimum detectable prey size for each species using both peak call frequency and maximum call frequency. The largest minimum detectable prey size estimate was found in omnivorous bats (*Phyllostomus hastatus* and *Phyllostomus discolor*), and the smallest minimum detectable prey size in insectivorous bats (Tables S2 and S3). In a phylogenetic ANOVA, we found no significant differences among detectable prey size estimates among dietary guilds (Figure 4; *p* > .10), although omnivores are significantly different from all other dietary guilds when phylogeny is not taken into account (prey size calculated with maximum frequency: *b* = 21 ± 7.20, *t* = 2.92, *p* = .0068; with peak frequency: *b* = 15.67 ± 7.54, *t* = 2.08, *p* = .0468). Animalivores have the largest variance in minimum prey size for emitted peak frequency, whereas insectivores show the largest variance in detectable prey size estimated from emitted maximum frequency (Table S3). For functionally defined dietary guilds, we found that predators of non‐mobile/non‐evasive prey and predators of mobile/evasive prey differ in minimum detectable prey size estimated based on maximum frequency emitted, albeit at a greater alpha value (*b* = −5.61 ± 3.01, *t* = −1.86, *p* = .071; Figure 4). Predators of mobile/evasive prey show the largest values and variance in detectable prey size for both peak and max frequency (Table S3).

## 4 DISCUSSION

Sensory specializations determine a species’ perceptual field and can underlie differences in resource use among taxa (Coombs et al., 1988; Denzinger & Schnitzler, 2013; Safi & Siemers, 2010; Valenta et al., 2013; Weinbeer & Kalko, 2007; Zimmermann et al., 2009). Phyllostomid bats are narrow‐space foragers (Wilson & Reeder, 2005) and acoustically constrained by short‐range detection in a highly cluttered acoustic space (Schnitzler & Kalko, 2001). They represent an adaptive radiation in which species share foraging habitats, so they are a valuable system for evaluating how evolutionary relatedness, body size, and dietary ecology contribute to echolocation signal design, and potentially niche partitioning in sympatric species. In this study, we found phyllostomid echolocation call characteristics reflect dietary ecology to some extent, and that forces other than dietary specialization, such as phylogeny and body size, also predict call similarities and divergence among species.

We found a weak yet significant negative relationship between maximum call frequency and body size. Since maximum frequency defines the upper limit of echolocation call capability, this result can be explained by a known relationship in which an increase in the linear size of sound‐producing structures results in lower frequencies (Pye, 1979). However, we did not find any scaling relationship between any other call parameter and body size. While a recent study of sympatric Amazonian phyllostomids found a negative relationship between peak frequency and body size (Yoh et al., 2020), our results largely corroborate Jones (1999) findings that phyllostomids diverge from the allometric pattern found in other bat families. Other morphological features, such as vocal tract geometry (Hartley & Suthers, 1988; Neuweiler, 2000) or nose leaf morphology (Hartley & Suthers, 1987; Leiser‐Miller & Santana, 2020; Vanderelst et al., 2010), might be better predictors of emitted frequency than body size in phyllostomids, as the geometry of sound‐producing structures can also influence the frequency emitted (Hartley & Suthers, 1988; Jakobsen et al., 2013; Neuweiler, 2000). For example, phyllostomid species with a coronally flattened nose leaf and a reduced ventral edge of the horseshoe have lower maximum frequencies in their echolocation calls (Leiser‐Miller & Santana, 2020). Moreover, given that phyllostomids use frequency‐modulated calls and can exploit a wide range of frequencies, this could relax constraints on the evolution of call parameters. That is, while some parameters (e.g., maximum frequency) may be more constrained by the physical limitations of sound production, others (e.g., peak frequency) may be more plastic to match tasks associated with foraging habitat or prey detection (Jacobs et al., 2007).

Consistent with our predictions, both taxonomic and functional dietary guilds differ in major parameters that define echolocation call structure. Call parameters were more effective at predicting functionally defined dietary guilds than taxonomically defined guilds; however, there was some overlap among categories. This suggests that call structure may be—to some extent—specialized for different types of food items, whereas call parameters may be more reflective of specialization on specific foraging behaviors necessary to capture the different prey types. For instance, higher call frequencies reduce detection distance (e.g., in species searching along leaf clutter for insects; e.g., *Micronycteris microtis*; Geipel et al., 2013) but allow perception of smaller prey (e.g., detection of small insects, fruits, or flowers). Conversely, lower frequencies allow for detection over longer ranges, but provide less resolution, which is only suitable for detecting larger prey (Fenton et al., 2016; Neuweiler, 2000). Based on our findings, these functional requirements of, and trade‐offs among, echolocation parameters may be more influential on call evolution than simple prey taxonomy. Even so, some species do not have the call structure that would be predicted for their dietary guild. This interesting finding suggests that more detailed, quantitative studies of foraging behavior and diet are still needed to further elucidate the relationship between call structure and dietary ecology in phyllostomids.

Both peak frequency and minimum frequency are primary drivers of the observed call differences among phyllostomid dietary guilds. Omnivorous phyllostomids have the lowest minimum and peak frequency and are the most distinct from other guilds. In other bat families, peak frequency and minimum frequency are important for distinguishing among species (Fenton & Bell, 1981; Hughes et al., 2011; Vaughan et al., 1997). According to our measurements, some phyllostomid species can also be distinguishable by the peak and minimum frequencies of their echolocation calls. This suggests that changes in most frequency‐linked call parameters may reflect species‐specific specialization for ecological niches; however, the total variation in call structure seen in phyllostomids cannot be fully explained by dietary niches as there is considerable overlap in calls among guilds.

Contrary to our predictions, time‐linked parameters (i.e., duration) did not differ among any of the dietary guilds, suggesting these may be more plastic among species than frequency‐linked parameters. This has been shown in some frugivorous phyllostomids (e.g., Leiser‐Miller et al., 2020) and species within other bat families that use time‐delayed information for localization of objects. Plasticity in time‐linked parameters may help mediate acoustic masking (i.e., masking by echoes from foliage or objects; Denzinger & Schnitzler, 2013) and navigate complex acoustic environments rapidly and with agility (Jones & Holderied, 2007; Moss & Surlykke, 2010; Schnitzler et al., 2003; Surlykke & Moss, 2000).

Acoustic detection of preferred prey size is constrained by wavelength and has only been studied in a few bat species. Thomas et al. (2004) found that species emitting the highest frequencies (shortest wavelengths) fed on the smallest insects. However, the species that emitted the lowest frequencies (longest wavelengths) fed on insects that were smaller than predicted by wavelength alone. We estimated the minimum detectable prey size across phyllostomid species and found no major differences among guilds when phylogeny is considered, but some guilds do exhibit greater variance than others in minimum detectable prey size estimates (i.e., animalivores, insectivores, predators of mobile/evasive prey). A substantial number of phyllostomid species feed on animal prey (Wilson & Reeder, 2005); therefore, a greater variance in detectable prey size may reflect both their phylogenetic (species) and ecological diversity. The variance in echolocation call design within guilds could further reflect dietary adaptation and niche partitioning through sensory biases. For instance, small differences in vespertilionid bats’ (insectivores) echolocation call structure contributes to niche differentiation within guilds (Siemers & Schnitzler, 2004; Siemers & Swift, 2006). Further research is needed to determine if phyllostomid echolocation signals reflect finer resolution differences in consumed taxa among species.

Phyllostomids have evolved other sensory specializations beyond echolocation, which they also use for food detection. For example, *Desmodus rotundus* (sanguinivore) uses infrared sensing pits to sense warm mammals (Jones et al., 2013) and *Trachops cirrhosus* and other animalivorous species use passive hearing to detect prey (Kalko et al., 1999). Many plant‐eating and omnivorous species use olfaction and vision and rely on a multimodal sensing approach for prey detection (Bell & Fenton, 1986; Kalko & Condon, 1998; Korine & Kalko, 2005; Leiser‐Miller et al., 2020; Thies et al., 1998). Alternative or complementary sensory modalities are expected to relax selection on echolocation call specialization, but it is still poorly understood how multimodal sensing plays into unique foraging scenarios in phyllostomids. Even though these bats are diverse in their sensory abilities, there is growing experimental evidence that phyllostomid species across dietary guilds use echolocation to find prey (Geipel et al., 2013; Gonzalez‐Terrazas, Koblitz, et al., 2016; Kalko & Condon, 1998; Thies et al., 1998). Therefore, the evolution of echolocation calls in the context of the phyllostomid dietary radiation likely involves a complex interaction with the evolution of other sensory modalities.

All phyllostomid species forage and/or have to navigate dense clutter (Schnitzler & Kalko, 2001), and the extreme acoustic characteristics of this habitat may impose strong evolutionary pressures on echolocation call structure (Broders et al., 2005; Denzinger & Schnitzler, 2013; Schnitzler & Kalko, 2001; Siemers & Schnitzler, 2000, 2004). Schnitzler et al. (2003) argued that echolocation call structure first evolved for spatial orientation and secondarily for prey acquisition. Under this scenario, because species that forage in similar habitats must solve similar tasks, they are expected to share sensory system characteristics, particularly in the design of echolocation call signals (Schnitzler et al., 2003). Therefore, habitat constraints likely explain the broad overlap in call design we report across phyllostomids species.

## 5 CONCLUSIONS

Our results suggest that phyllostomids have more diverse echolocation calls than previously reported. While their call structure may be primarily adapted for dealing with acoustic constraints of foraging in dense habitats, it appears to be secondarily specialized to some extent for detection of food items across major dietary guilds. Further research on multimodal sensing, prey detection behavior, and greater knowledge of species’ dietary ecology will help further understand differences in echolocation call design in the phyllostomid adaptive radiation. We hope the detailed information presented here on the echolocation calls of a representative sample of phyllostomids can serve as the basis of future studies aiming to more broadly understand the functionality of bat echolocation systems.

## CONFLICT OF INTEREST

None declared.

## AUTHOR CONTRIBUTIONS

**Leith B. Leiser‐Miller:** Conceptualization (equal); Data curation (lead); Formal analysis (lead); Investigation (lead); Methodology (equal). **Sharlene E. Santana:** Conceptualization (equal); Data curation (supporting); Formal analysis (supporting); Funding acquisition (lead); Investigation (equal); Methodology (equal).

## Supporting information

Appendix S1

## Data Availability

All data used in analyses are included in the Appendix S1.
