# Supplementary material for: Functional differences in echolocation call design in an adaptive radiation of bats
Source: Ecol Evol. 2021 Nov 2;11(22):16153–64. doi: 10.1002/ece3.8296 (PMC8601877; doi:10.1002/ece3.8296)
Supplement: Supplementary file 1 — Appendix S1 [file ECE3-11-16153-s001.docx]

Supplementary Information for

**Functional differences in echolocation call design in an adaptive radiation of bats**

**TABLES**

**Table S1.** Call parameter means, standard deviations (SD) and range (minimum - maximum values, listed beneath) for duration (dur.), maximum frequency (maxF), minimum frequency (minF), peak frequency (pF), bandwidth (bw), and harmonics (harm.) across 35 species of phyllostomid bats.

**Table S2.** Summary of species, diet and calculated minimum detectable prey size based on peak frequency, pF (mm), and maximum frequency emitted, mF (mm).

**Table S3.** Group means, standard deviation (SD), and variance for minimum detectable prey size for taxonomic and functional dietary guilds, calculated from peak frequency (pF) and maximum frequency (mF).

**FIGURES**

**Figure S1:** Phylogenetic generalized least squares regression of maximum frequency on

forearm length.

**Table S1.** Call parameter means, standard deviations (SD) and range (minimum - maximum values, listed beneath) for duration (dur.), maximum frequency (maxF), minimum frequency (minF), peak frequency (pF), bandwidth (bw), and harmonics (harm.) across 35 species of phyllostomid bats.

| Species | N | dur.(ms) | maxF (kHz) | minF (kHz) | peakF (kHz) | bw (kHz) | harm. |
| --- | --- | --- | --- | --- | --- | --- | --- |
| *Artibeus jamaicensis* | 8 | 0.78 ±0.42 | 93.1 ±3.7 | 63.2 ±6.1 | 77.1 ±4.4 | 39.3 ±12.6 | 1.54 ±0.27 |
|  |  | (0.042-1.5) | (83.4-96.0) | (53.1-71.7) | (69.1-82.5) | (25.6-58.5) | (1.10-1.90) |
| *Artibeus lituratus* | 4 | 0.84 ±0.15 | 91.5±3.49 | 57.7 ±4.23 | 72.9±1.7 | 34.8±5.8 | 1.33 ±0.33 |
|  |  | (0.61-0.95) | (88.3-95.2) | (52.4 -61.6) | (71.8-75.5) | (27.3-40.6) | (1.0-1.80) |
| *Carollia castanea* | 7 | 0.44 ±0.09 | 110.0 ±4.29 | 56.0 ±5.27 | 80.7 ±4.2 | 58.1 ±9.7 | 1.35 ±0.345 |
|  |  | (0.31-0.54) | (115.7-104.4) | (47.9 -64.1) | (73.5-86.4) | (39.7-68.0) | (1.00-1.80) |
| *Carollia perspicillata* | 12 | 0.65 ±0.30 | 98.4 ±11.6 | 56.8 ± 11.9 | 77.7 ±7.3 | 48.8 ±15.2 | 1.47 ± 0.23 |
|  |  | (0.29-1.11) | (84.8-124.2) | (29.7-74.8) | (68.1-88.4) | (29.9-87.7) | (1.2-1.86) |
| *Carollia sowelli* | 11 | 0.57 ±0.24 | 94.7 ±14.7 | 64.5 ±17.1 | 78.8 ±15.4 | 33.8 ±5.4 | 1.41 ±0.13 |
|  |  | (0.28-1.00) | (85.0-137.2) | (41.6-107.1) | (65.4-121.8) | (25.1-44.8) | (1.10-1.60) |
| *Carollia subrufa* | 4 | 0.52 ±0.12 | 100.6 ±1.6 | 64.6 ±6.8 | 85.5 ±2.9 | 37.5 ±1.1 | 1.29 ±0.14 |
|  |  | (0.42-0.66) | (49.3-102.9) | (55.2-70.6) | (82.4-89.5) | (36.4-38.6) | (1.10-1.40) |
| *Centurio senex* | 5 | 1.04 ±0.22 | 122.6 ±12.5 | 67.8 ±3.1 | 86.7 ±4.1 | 34.5 ±7.3 | 1.05 ±0.08 |
|  |  | (0.93-1.37) | (106.1-141.3) | (63.7-71.4) | (83.5-94.0) | (25.7-42.3) | (1.00-1.21) |
| *Chrotopterus auritus* | 1 | 0.28 | 139.1 | 96.3 | 118.2 | 42.1 | 2.47 |
|  |  | - | - | - | - | - | - |
| *Dermanura phaeotis* | 10 | 0.53 ±0.08 | 105.0 ±8.9 | 59.8 ±5.5 | 81.3 ±7.9 | 50.2 ±9.7 | 1.48  ±0.36 |
|  |  | (0.43-0.70) | (84.9-114.2) | (49.7-68.2) | (72.4-90.1) | (38.6-70.4) | (1.05-2.36) |
| *Dermanura watsoni* | 6 | 0.45 ± 0.10 | 115.1 ± 4.8 | 63.1 ±10.4 | 91.9 ±7.1 | 50.7 ±12.1 | 1.48 ± 0.17 |
|  |  | (0.28-0.57) | (109.1-120.4) | (55.3-80.2) | (78.5-99.8) | (30.6-64.8) | (1.33-1.73) |
| *Desmodus rotundus* | 3 | 0.74 ±0.18 | 89.8 ±7.7 | 64.4 ±1.7 | 78.1 ±3.7 | 30.7 ±1.8 | 1.18 ±0.03 |
|  |  | (0.61-0.95) | (81.1-96.2) | (62.4-65.8) | (73.9-80.9) | (29.1-32.7) | (1.13-1.21) |
| *Ectophylla alba* | 5 | 0.63 ±0.11 | 128.9 ±10.4 | 49.1 ±8.8 | 81.1 ±10.7 | 71.4 ±15.4 | 1.27 ± 0.16 |
|  |  | (0.46-0.74) | (118.4-144.5) | (36.6-59.1) | (69.7-93.7) | (56.1-95.3) | (1.07-1.5) |
| *Gardnerycteris crenulatum* | 11 | 0.53 ±0.26 | 84.0 ±3.7 | 56.8 ±2.2 | 66.5 ±1.8 | 24.6 ±4.6 | 1.31 ±0.15 |
|  |  | (0.38-1.32) | (80.1-93.3) | (53.6-61.9) | (63.4-69.3) | (18.8-35.6) | (1.09-1.50) |
| *Glossophaga commissarisi* | 4 | 0.86 ±0.51 | 124.4 ±24.1 | 49.5 ±16.7 | 81.0 ±7.1 | 81.3 ±30.7 | 1.54 ±0.39 |
|  |  | (0.33-1.48) | (91.8-149.9) | (34.2-71.9) | (72.3-89.8) | (43.0-114.3) | (1.23-1.91) |
| *Glossophaga leachii* | 1 | 0.45 | 135.0 | 61.3 | 91.6 | 84.1 | 1.60 |
|  |  | - | - | - | - | - | - |
| *Glossophaga soricina* | 4 | 0.58 ±0.27 | 117.5 ±6.1 | 54.5 ±19.5 | 82.6 ±12.3 | 75.2 ±17.0 | 1.38 ±0.12 |
|  |  | (0.31-0.95) | (111.9-124.4) | (32.8-78.6) | (67.9-97.3) | (60.1-97.4) | (1.21-1.50) |
| *Glyphonycteris sylvestris* | 1 | 1.49 | 92.2 | 51.0 | 70.8 | 41.1 | 2.80 |
|  |  | - | - | - | - | - | - |
| *Hylonycteris underwoodii* | 2 | 0.36 ±0.14 | 110.4 ±19.8 | 49.4 ±3.3 | 78.2 ±14.7 | 56.1 ±30.3 | 1.49 ±0.51 |
|  |  | (0.26-0.46) | (96.3-124.5) | (47.0-51.8) | (67.8-88.6) | (34.7-77.6) | (1.12-1.85) |
| *Lampronycteris brachyotis* | 8 | 0.53 ±0.12 | 135.6 ±10.5 | 66.0 ±16.9 | 102.5 ±10.0 | 68.4 ±15.3 | 1.80 ±0.49 |
|  |  | (0.38-0.60) | (116.8-161.9) | (42.0-89.0) | (80.4-116.6) | (39.6-103.8) | (1.10-2.53) |
| *Lophostoma brasiliense* | 5 | 0.44 ±0.81 | 121.2 ±7.4 | 59.1 ±11.5 | 88.3 ± 12.9 | 64.3 ±10.9 | 2.08 ±0.15 |
|  |  | (0.36-0.58) | (113.1-133.4) | (45.9-73.8) | (70.6-100.6) | (48.0-77.9) | (1.86-2.28) |
| *Lophostoma silvicolum* | 2 | 0.79 ±0.17 | 85.7 ±15.3 | 56.8 ±0.56 | 69.5 ±8.2 | 33.6 ±12.8 | 1.40 ±0.49 |
|  |  | (0.67-0.92) | (74.9-96.6) | (56.4-57.2) | (63.7-75.3) | (24.5-42.7) | (1.05-1.76) |
| *Mesophylla macconnelli* | 1 | 0.63 | 134.2 | 84.0 | 111.6 | 43.0 | 1.69 |
|  |  | - | - | - | - | - | - |
| *Micronycteris hirsuta* | 4 | 0.40 ±0.13 | 101.7 ±9.1 | 57.5 ±7.9 | 76.7 ±6.6 | 41.4 ±10.9 | 1.83 ±0.22 |
|  |  | (0.22-0.54) | (89.2-108.7) | (50.1-68.6) | (70.9-85.5) | (30.6-51.5) | (1.53-2.07) |
| *Micronycteris microtis* | 3 | 0.35 ±.073 | 105.1 ±8.3 | 59.7 ±9.8 | 79.4 ±2.4 | 9.15 ±20.33 | 2.02 ±0.98 |
|  |  | (0.29-0.43) | (96.0-112.4) | (48.8-67.6) | (77.1-81.9) | (39.1-55.2) | (1.45-3.15) |
| *Micronycteris schmidtorum* | 1 | 0.34 | 117.2 | 52.2 | 83.7 | 65.2 | 1.96 |
|  |  | - | - | - | - | - | - |
| *Phyllostomus discolor* | 6 | 0.83 ±0.21 | 68.2 ±9.8 | 41.1 ±4.0 | 54.3 ±8.5 | 33.6 ±4.0 | 1.39 ±0.21 |
|  |  | (0.58-1.16) | (56.8-79.5) | (35.8-46.0) | (45.8-64.7) | (28.0-38.2) | (1.08-1.63) |
| *Phyllostomus hastatus* | 1 | 0.74 | 63.6 | 21.3 | 54.3 | 56.3 | 2.3 |
|  |  | - | - | - | - | - | - |
| *Platyrrhinus helleri* | 2 | 0.74 ±0.16 | 119.2 ±1.1 | 75.6 ±1.8 | 102.4 ±0.02 | 45.3 ±8.8 | 1.34 ±0.22 |
|  |  | (0.62-0.86) | (118.3-120.0) | (74.3-76.9) | (102.4-102.5) | (39.0-51.6) | (1.18-1.50) |
| *Sturnira lilium* | 3 | 0.54 ±0.12 | 119.9 ±16.2 | 62.4 ±14.9 | 89.1 ±1.1 | 53.0 ±25.2 | 1.51  ±0.18 |
|  |  | (0.45-0.69) | (101.2-131.0) | (47.2-77.2) | (88.4-90.4) | (24.8-73.4) | (1.38-1.72) |
| *Tonatia bidens* | 3 | 0.62 ±0.15 | 82.0 ±6.4 | 53.6 ±5.8 | 68.2 ±5.4 | 35.6 ±6.7 | 1.25 ±0.08 |
|  |  | (0.52-0.83) | (75.5-88.4) | (49.9-60.3) | (62.0-72.5) | (28.6-41.9) | (1.20-1.35) |
| *Tonatia saurophila* | 4 | 0.86±0.28 | 82.7 ±10.0 | 53.5 ±3.8 | 66.6 ±5.8 | 34.2 ±5.4 | 1.39 ±0.25 |
|  |  | (0.71-1.29) | (73.5-97.0) | (48.3-57.7) | (58.6-72.7) | (29.5-41.3) | (1.17-167) |
| *Trachops cirrhosus* | 7 | 0.58 ±0.23 | 109.7 ±11.4 | 40.5 ±13.9 | 74.7 ±12.8 | 74.2 ±20.9 | 1.77 ±0.44 |
|  |  | (0.33-0.99) | (92.8-122.6) | (24.9-63.2) | (58.7-93.4) | (39.3-97.0) | (1.07-2.29) |
| *Vampyriscus nymphaea* | 2 | 0.57±0.13 | 116.6 ±14.5 | 72.6 ±3.0 | 91.4 ±11.9 | 55.5 ±7.4 | 1.57 ±0.24 |
|  |  | (0.48-0.67) | (106.3-126.9) | (70.5-74.8) | (82.9-99.8) | (50.3-60.8) | (1.40-1.74) |
| *Vampyrum spectrum* | 1 | 0.52 ±0.08 | 103.5 ±2.8 | 64.0 ±1.5 | 83.0 ±0.7 | 32.8 ±16.0 | 1.23 ±0.32 |
|  |  | - | - | - | - | - | - |

**Table S2.** Summary of species, diet and calculated minimum detectable prey size based on peak frequency, peakF (mm), and maximum frequency emitted, maxF (mm).

| **Species** | **Diet** | **peakF (mm)<** | **maxF (mm)<** |
| --- | --- | --- | --- |
| Artibeus jamaicensis | fruit | 4.52 | 3.89 |
| Artibeus lituratus | fruit | 4.76 | 4.06 |
| Carollia castanea | fruit | 4.30 | 3.24 |
| Carollia perspicillata | fruit | 4.47 | 3.54 |
| Carollia sowelli | fruit | 4.41 | 3.81 |
| Carollia subrufa | fruit | 4.05 | 3.47 |
| Centurio senex | fruit | 4.00 | 3.63 |
| Chrotopterus auritus | animal | 2.49 | 2.94 |
| Dermanura phaeotis | fruit | 4.21 | 3.40 |
| Dermanura watsoni | fruit | 3.77 | 3.17 |
| Desmodus rotundus | blood | 4.44 | 3.96 |
| Ectophylla alba | fruit | 4.28 | 2.90 |
| Gardnerycteris crenulatum | insect | 5.21 | 4.33 |
| Glossophaga commissarisi | nectar | 4.28 | 2.90 |
| Glossophaga leachii | nectar | 3.79 | 2.74 |
| Glossophaga soricina | nectar | 4.11 | 2.95 |
| Glyphonycteris sylvestris | insect | 3.76 | 4.90 |
| Hylonycteris underwoodi | nectar | 4.44 | 3.38 |
| Lampronycteris brachyotis | insect | 3.38 | 2.76 |
| Lophostoma brasiliense | insect | 3.93 | 2.90 |
| Lophostoma silvicolum | insect | 4.99 | 4.02 |
| Mesophylla macconnelli | fruit | 3.11 | 2.70 |
| Micronycteris hirsuta | insect | 4.52 | 3.56 |
| Micronycteris microtis | insect | 4.37 | 3.35 |
| Micronycteris minuta | insect | 4.00 | 3.61 |
| Micronycteris schmidtorum | insect | 4.14 | 2.96 |
| Phyllostomus discolor | omnivore | 6.38 | 4.95 |
| Phyllostomus hastatus | omnivore | 6.38 | 4.73 |
| Platyrrhinus helleri | fruit | 3.39 | 2.95 |
| Sturnira lilium | fruit | 3.89 | 3.09 |
| Tonatia bidens | insect | 5.08 | 4.16 |
| Tonatia saurophila | insect | 5.21 | 4.22 |
| Trachops cirrhosus | animal | 4.64 | 3.20 |
| Vampyriscus nymphaea | fruit | 3.80 | 3.17 |
| Vampyrum spectrum | animal | 4.15 | 3.34 |

**Table S3.** Group means, standard deviation (SD), and variance for minimum detectable prey size for taxonomic and functional dietary guilds, calculated from peak frequency (peakF) and maximum frequency (maxF).

| Prey size variable | Diet | Mean ± SD | Variance |
| --- | --- | --- | --- |
| Min.(peakF) | animal | 3.76 ± 1.12 | 1.26 |
|  | blood | 4.44 ± NA | NA |
|  | fruit | 4.06 ± 0.45 | 0.20 |
|  | insect | 4.41 ± 0.60 | 0.36 |
|  | nectar | 4.05 ± 0.25 | 0.06 |
|  | omnivore | 6.38 ± 9.4e-4 | 8.8e-7 |
| Min.(maxF) | animal | 3.15 ± 0.20 | 0.04 |
|  | blood | 3.95 ± NA | NA |
|  | fruit | 3.35 ± 0.39 | 0.15 |
|  | insect | 3.67 ± 0.65 | 0.43 |
|  | nectar | 2.86 ± 0.10 | 0.01 |
|  | omnivore | 4.84 ± 0.15 | 0.02 |
| Min.(peakF) | non-mobile/non-evasive | 4.08 ± 0.41 | 0.17 |
|  | mobile/evasive | 4.53 ± 0.97 | 0.95 |
| Min.(maxF) | non-mobile/non-evasive | 3.30 ± 0.42 | 0.18 |
|  | mobile/evasive | 3.72 ± 0.72 | 0.52 |


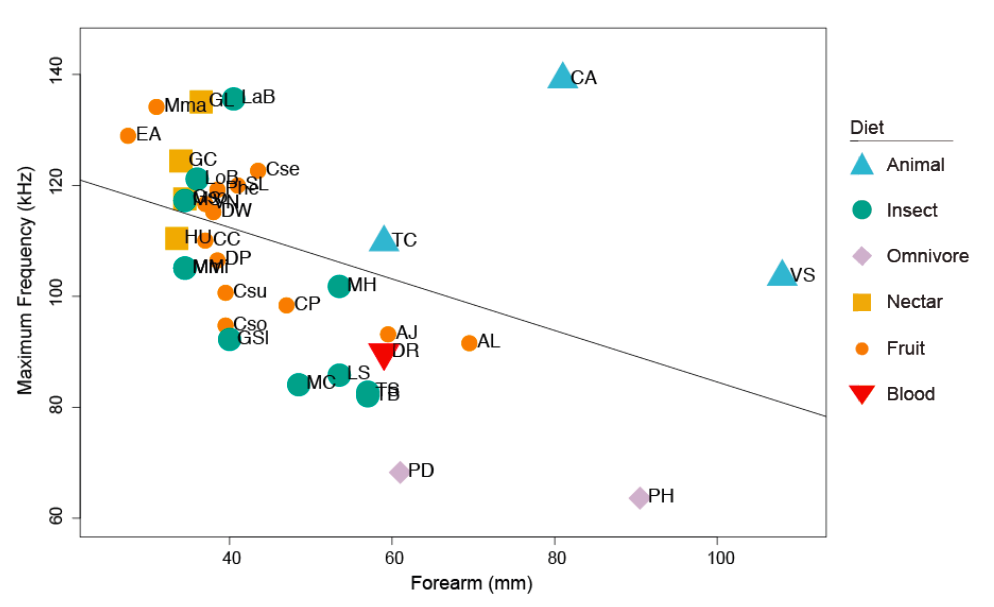


**Figure S1:** Phylogenetic generalized least squares regression of maximum frequency on

forearm length, MaxFreq = -0.4766FL +129.24, R^2^=0.2. Species codes are listed as AJ-*Artibeus jamaicensis,* AL*-Artibeus lituratus,* CC*-Carollia castanea,* CP*-Carollia perspicillata,* Cso-*Carollia sowelli,* Csu*-Carollia subrufa,* Cse-*Centurio senex,* CA*-Chrotopterus auritus,* DP-*Dermanura phaeotis,* DW*-Dermanura watsonii,* DR*-Desmodus rotundus,* EA*-Ectophylla alba,* GC*-Glossophaga commissarisi,* GL*-Glossophaga longirostirs,* Gso*-Glossophaga soricina,* GSl*-* *Glyphonycteris sylvestris,* HU*-Hylonycteris underwoodi,* LaB*-Lampronycteris brachyotis,* LoB - *Lophostoma brasiliense,* LS*-Lophostoma silvicolum,* Mma*-Mesophylla macconnelli,* MH -

*Micronycteris hirsuta,* MM*-Micronycteris microtis,* Mmi*-Micronycteris minuta,* MS-*Micronycteris schmidtorum,* MC*-Gardnerycteris crenulatum,* PD*-Phyllostomus discolor,* PH-

*Phyllostomus hastatus,* Phe*-Platyrrhinus helleri,* SL*-Sturnira lilium,* TB*-Tonatia bidens,* TS-*Tonatia saurophila,* TC*-Trachops cirrhosus,* VN*-Vampyriscus nymphaea,* VS*-Vampyrum spectrum.*
